# Comparison of the PK, PD, safety, tolerability, and immunogenicity of proposed biosimilar RGB-19 and tocilizumab in healthy Japanese males: a phase 1, randomised, crossover study

**DOI:** 10.1016/j.ero.2025.08.006

**Published:** 2025-09-24

**Authors:** Tomoko Hasunuma, Paul Emery, Ernest Choy, Masato Okada, Roshan Dias, Károly Horvát-Karajz, Gordana Dancer, Attila Kónya, Yusuke Karibe, Kazuya Uchida, Suguru Masuda, Joachim Kiefer, Gerd R. Burmester

**Affiliations:** 1 Department of Research, Kitasato University, Kitasato Institute Hospital, Tokyo, Japan; 2 NIHR Biomedical Research Centre, Leeds Teaching Hospitals Trust and Leeds Institute of Rheumatic and Musculoskeletal Medicine, University of Leeds, Leeds, UK; 3 Division of Infection and Immunity, CREATE Centre, Cardiff University, Cardiff, UK; 4 Immuno-Rheumatology Centre, St. Luke's International Hospital, Tokyo, Japan; 5 Gedeon Richter Plc, Budapest, Hungary; 6 Mochida Pharmaceutical Co., Ltd, Tokyo, Japan; 7 Department of Rheumatology and Clinical Immunology, Charité—Universitätsmedizin Berlin, Germany

## Abstract

**Objectives:**

RGB-19 is a proposed biosimilar to tocilizumab. This phase 1 study assessed the equivalence in pharmacokinetics (PKs) and compared the pharmacodynamics (PDs), safety, and immunogenicity of a single subcutaneous administration of RGB-19 and tocilizumab.

**Methods:**

Healthy Japanese males aged 20 to 40 years were included in this 2-period, 2-sequence crossover study. Participants were randomised 1:1 to RGB-19 162 mg or tocilizumab 162 mg in period 1 (day -1 to day 42), before crossing over treatment in period 2 (day 42 to day 85). The primary objective was to assess equivalence between treatments in the maximum serum drug concentration (*C*_max_) and area under the serum drug concentration curve from 0 hours to infinity (AUC_inf_) using predefined 2-sided 90% CI criteria of 0.80 to 1.25. Secondary objectives included assessment of PK, PD, safety, tolerability, and immunogenicity.

**Results:**

In period 1, 110 participants were included, followed by 102 participants in period 2. PK equivalence was demonstrated between treatments for *C*_max_ and AUC_inf_ (*C*_max_ point estimate: 1.04 [90% CI: 0.98-1.10]; AUC_inf_ point estimate: 1.05 [90% CI: 0.97-1.13]). Concentration-time profiles for PD parameters were similar between treatments. Antidrug antibody (ADA) and neutralising antibody incidences were similar between treatments, and ADA status had no impact on PK, PD, or safety outcomes. There were no notable differences in treatment-emergent adverse events or adverse drug reactions.

**Conclusions:**

This study demonstrated equivalence in PK and similarity in PD, immunogenicity, and safety of RGB-19 and tocilizumab. These data suggest RGB-19 provides a similar therapeutic benefit to tocilizumab.

**WHAT IS ALREADY KNOWN ON THIS TOPIC**

• Biologics such as tocilizumab are vital treatment options for many diseases, but high treatment costs may limit patient access. Biosimilars may provide a cheaper alternative.

**WHAT THIS STUDY ADDS**

• This study demonstrates the equivalence in pharmacokinetics and similarity in pharmacodynamics, safety, and immunogenicity of the proposed biosimilar RGB-19 and tocilizumab.

**HOW THIS STUDY MIGHT AFFECT RESEARCH, PRACTICE, OR POLICY**

• These results provide data supporting the biosimilarity of RGB-19 and tocilizumab; if approved, RGB-19 may prove to be a cheaper alternative to licensed tocilizumab with a similar therapeutic effect.

Alt-text: Unlabelled box

## INTRODUCTION

Interleukin (IL)-6 is an inflammatory cytokine that plays a key role in a number of inflammatory diseases, including rheumatoid arthritis (RA), through activation of T-cells and induction of immunoglobulin secretion, among other physiological processes [1,2]. The introduction of biological disease-modifying antirheumatic drugs significantly changed RA treatment, with IL-6 inhibitors shown to be efficacious in patients with an inadequate response to methotrexate [3].

Tocilizumab is a humanised antihuman IL-6 receptor (IL-6R) antibody that acts by competitively inhibiting IL-6 signalling by blocking the IL-6 binding site of IL-6R [4,5]. This leads to normalisation of markers of inflammation, including acute phase proteins and C-reactive protein (CRP) [5]. The effectiveness [[6], [7], [8], [9], [10], [11], [12], [13], [14]] and tolerability [11,15,16] of tocilizumab in patients with RA have been well established in numerous studies. It was approved for the treatment of RA in Japan in 2008 [17], Europe in 2009 [18], and the USA in 2010 [19]. It is available as both intravenous (IV) and subcutaneous (SC) formulations, with the latter permitting self-administration [18].

Biologics such as tocilizumab have been shown to be an effective treatment option in numerous diseases; however, they can be associated with high costs that may limit patient access [20,21]. Biosimilars may provide a cheaper alternative to originator products [22,23]. As such, tocilizumab biosimilars have the potential to widen access to people with diseases such as RA.

RGB-19 is a proposed biosimilar to tocilizumab with the same dosage form, strength, and route of administration as originator tocilizumab [18,24]. The functional similarity between RGB-19 and tocilizumab has been demonstrated previously [25]. This phase 1 study (jRCT2031230029) investigated the equivalence in pharmacokinetics (PKs) of SC RGB-19 and tocilizumab and compared the pharmacodynamics (PDs) and safety, including immunogenicity, of a single SC administration of each treatment in healthy adult men.

## METHODS

### Study design

**Figure 1:**
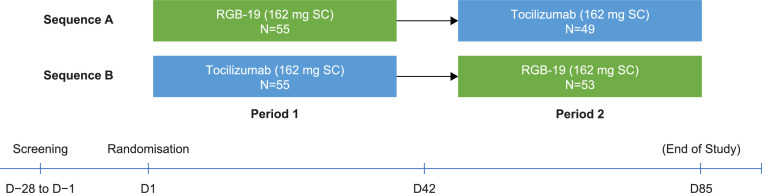
Study design. D, day; SC, subcutaneous.

This was a phase 1, randomised, double-blind, 2-treatment, 2-period, 2-sequence crossover study (Fig 1). Participants were randomised 1:1 to a single SC injection of RGB-19 162 mg or EU-licensed tocilizumab (hereafter tocilizumab) 162 mg, administered on day 1 in period 1 (day −1 to day 42). Participants then crossed over to the other treatment on day 43 (period 2 [day 42 to day 85]). Participants in sequence A received RGB-19 in period 1, then switched to tocilizumab in period 2, whilst participants in sequence B received tocilizumab in period 1, then RGB-19 in period 2. Participants were randomly assigned to a sequence through allocation tables using random numbers. In periods 1 and 2, the investigational products (IPs) were allocated by at least 2 persons in a secure area where only unblinded personnel had access. The packaging was labelled to enable tracing of IP to each subject, and administrations were performed on a drug-by-drug basis. No medical treatment was planned after day 85 (study end); however, additional follow-up was conducted in the case of unresolved serious adverse events (AEs) or treatment-related AEs.

### Participant population

Participants were healthy Japanese male volunteers aged 20 to 40 years with a body mass index of ≥18.5 kg/m^2^ and <25.0 kg/m^2^. Participants’ body weight was required to be between ≥50 kg and <80 kg at screening.

Participants were excluded if they met any of the following criteria: previously exposed to tocilizumab or any other IL-6 or IL-6R inhibitor; abnormal values in haematology tests, blood coagulation tests, or urinalysis during screening; a positive hepatitis B/C, syphilis or HIV test result; receipt of a live vaccine 12 weeks prior to IP administration in period 1 or were scheduled to receive a live vaccine during the study period; or receipt of another IP within 16 weeks or 5 half-lives of the IP, whichever was longer, prior to the day of IP administration in period 1. Participants were also excluded if they had a current serious infectious disease or a history of serious infectious disease, chronic or recurrent infections requiring treatment with antiinfective drugs or hospitalisation. Full exclusion criteria are provided in the supplementary material (Supplementary methods).

### Patient and public involvement statement

Patients or the public were not involved in the design, conduction, reporting, and dissemination plans of this research.

### Study objectives and endpoints

The primary objective was to assess the equivalence of serum drug concentration of RGB-19 with tocilizumab; primary endpoints were maximum serum drug concentration (*C*_max_) and area under the curve (AUC) from 0 hours (immediately before administration) to infinity (AUC_inf_).

Secondary objectives were to evaluate additional PK parameters, via the following endpoints: AUC from 0 hours (immediately before administration) to the last quantifiable time (*t*) (AUC_last_), AUC from 0 hours (immediately before administration) to 144 hours after administration (AUC_0-144_), AUC from 144 hours after administration to the last quantifiable t (AUC_144-t_), time to maximum serum concentration (*t*_max_), elimination half-life (*t*_1/2_), apparent volume of distribution (*V*_d_/F), apparent total clearance (CL/F), and elimination rate constant (*k*_el_). Another secondary objective was to assess PD markers via the following endpoints: absolute neutrophil count (ANC), high-sensitivity CRP (hsCRP), and soluble IL-6R (sIL-6R). Safety (monitor AEs, vital signs, physical examination, injection site reactions, body weight, laboratory tests, and electrocardiogram) and immunogenicity (antidrug antibody [ADA] positivity, titre, and neutralising activity) were also evaluated as secondary endpoints. A subgroup analysis was conducted to assess the impact of ADAs on the primary PK parameters.

Serum drug concentration and PD outcomes were measured by validated immunoassay with electrochemiluminescent detection using blood serum sampled in each period at 24 time points: before IP administration and 6, 12, 24, 36, 48, 60, 72, 84, 96, 108, 120, 132, 144, 168, 192, 240, 288, 336, 408, 480, 576, 840, and 1008 hours after IP administration. Samples taken predose and on days 13, 43, 55, and 85 were tested by electrochemiluminescence immunoassay for the presence or absence of ADA to evaluate immunogenicity. Confirmed ADA-positive samples were further tested to determine the ADA titre and to detect antidrug neutralising antibodies (NAb).

An AE occurring on or after the first treatment administration was considered a treatment-emergent adverse event (TEAE). Adverse drug reactions (ADRs) were defined as treatment-related TEAEs. AEs were tabulated using the preferred terms of Medical Dictionary for Regulatory Activities/Japanese translation v27.0.

### Statistical analysis

Forty-four participants per treatment were estimated to be required to provide 90% power to assess the bioequivalence acceptance criteria of log (0.80) to log (1.25). A target sample size of 110 subjects was selected (55 per randomised sequence) to account for a 20% reduction in the number of participants included in the primary analysis due to discontinuation or dropout.

Statistical analysis was performed using SAS v9.4. PK parameters were calculated using noncompartmental methods with Phoenix WinNonlin v8.3. Populations were divided into PK, PD, safety, and immunogenicity analysis sets. The PK analysis set included participants who received the full dose of IP, could have either *C*_max_ or AUC_inf_ calculated, and had no major protocol deviations that could impact PK results. This threshold had to be met for both period 1 and period 2. The PD analysis set included participants who received the full IP dose, could have any PD parameter for ANC, hsCRP or sIL-6R calculated, and had no major protocol deviations that could impact PD results. This threshold had to be met for both period 1 and period 2. The safety analysis set included patients who were administered at least one (full or partial dose of) IP and had safety assessment data. The immunogenicity analysis set included patients who were administered at least one (full or partial dose of) IP, had a predose immunogenicity result and at least one available postdose immunogenicity assessment, and had no major protocol deviations that could impact immunogenicity results.

For the analysis of demographic data, continuous variables were assessed using descriptive statistics per allocation sequence; for binary variables, the number and percentage of participants were calculated by allocation sequence. Data for other endpoints were analysed by treatment.

To evaluate primary PK parameters, summary statistics were calculated for *C*_max_ and AUC_inf_ of serum drug concentrations for each participant and presented by treatment for the overall study period (period 1 and period 2). The difference between the natural log-transformed mean value of *C*_max_ and AUC_inf_ of serum drug concentration for each treatment and their two-sided 90% CI was calculated using an analysis of variance model with allocation sequence, participants, administration period, and treatment as fixed effects. Equivalence was demonstrated between the 2 treatments if the two-sided 90% CI for geometric mean ratio (GMR) of the *C*_max_ and AUC_inf_ of drug serum concentration met the predefined equivalence threshold of 0.80 to 1.25. The estimated means, difference of the means on a log-transformed scale, and the two-sided 90% CIs were calculated.

For the secondary PK parameters, descriptive statistics were calculated by treatment. The 2 treatments were compared with respect to all parameters using the same analysis of variance as for the primary PK parameters. All parameters were assumed to be log-normal distributed, except for the *t*_max_. For evaluation of PD, the measurement values and change from baseline in ANC, hsCRP, and sIL-6R were summarised by treatment, and the area under the effect-time curve (AUEC) of each PD marker was calculated. The number and percentage of ADA- and NAb-positive participants were summarised by allocation sequence, time point, and treatment. The results from the two-treatment periods were then combined. To evaluate safety, all AEs and TEAEs were summarised by the number of events and the number and percentage of participants (incidence), stratified by treatment.

### Ethics

The study protocol was reviewed by the institutional review board before the start of the study. The conduct of this clinical study met all local legal and regulatory requirements and was conducted in line with the Declaration of Helsinki. Written informed consent was obtained from each participant.

## RESULTS

### Participant population

A total of 110 participants were randomised in period 1 and received IP (sequences A and B, *n* = 55 each; Supplementary Fig S1). In period 2, a total of 102 participants received IP (sequence A, *n* = 49; sequence B, *n* = 53). In total, 8 (7.3%) participants prematurely withdrew from the study, all occurring during period 1. In sequence A (RGB-19), 6 (6/55; 10.9%) participants withdrew: 3 (3/55; 5.5%) due to AEs, 2 (2/55; 3.6%) of which were ADRs, and 3 (3/55; 5.5%) due to ‘other reasons (positive smoking test result)’. Two participants withdrew in sequence B (tocilizumab), 1 due to ‘a positive drug or alcohol test’ and 1 due to ‘other reasons (positive smoking test result)’.

Baseline demographics were similar between the 2 treatment groups (Table 1). Overall, all participants were of Asian race, 11 (10.0%) participants were in receipt of concomitant medications, and 1 (0.9%) participant had a concomitant procedure. In all participants, the reason for concomitant medications and the concomitant procedure was to treat or prevent AEs (for a list of treatments, see Supplementary results).

**Table 1:** Baseline demographics from participants administered investigational product (*N* = 110)

Characteristics	Allocation sequence		
	Sequence A: RGB-19—tocilizumab (*N* = 55)	Sequence B: tocilizumab—RGB-19 (*N* = 55)	Total (*N* = 110)
Age (y)			
Mean (SD)	28.4 (6.0)	30.6 (6.2)	29.5 (6.2)
Race, *n* (%)			
American Indian or Alaska Native	0 (0.0)	0 (0.0)	0 (0.0)
Asian	55 (100.0)	55 (100.0)	110 (100.0)
Black or African American	0 (0.0)	0 (0.0)	0 (0.0)
Native Hawaiian or other Pacific Islander	0 (0.0)	0 (0.0)	0 (0.0)
White	0 (0.0)	0 (0.0)	0 (0.0)
Height (cm)			
Mean (SD)	171.44 (5.24)	170.05 (5.27)	170.75 (5.28)
Body weight (kg) (day −1)			
Mean (SD)	62.75 (5.49)	61.07 (6.87)	61.91 (6.25)
BMI (kg/m^2^)			
Mean (SD)	21.37 (1.87)	21.08 (1.72)	21.22 (1.79)

BMI, body mass index; *N*, number of participants; *n*, number of participants for category; SD, standard deviation.

### PKs

**Figure 2:**
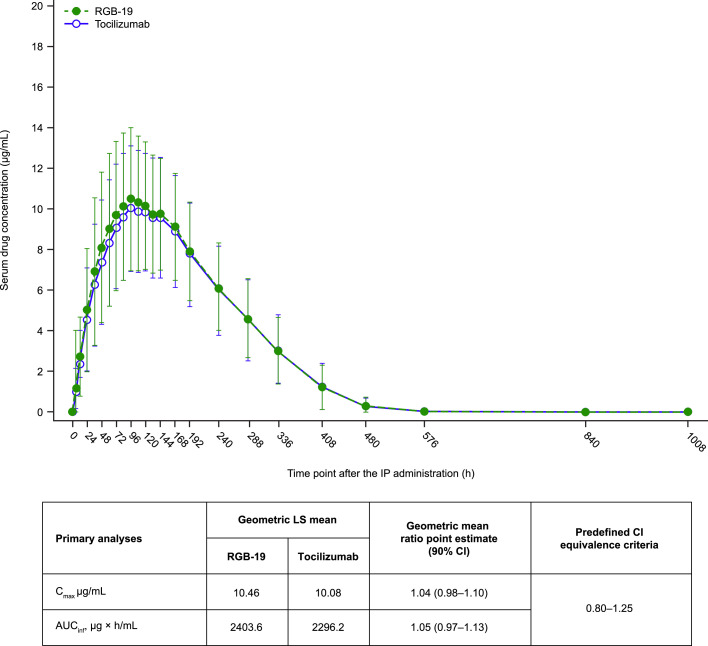
Mean (SD) serum drug concentration over time in the PK analysis set (*N* = 102) and geometric mean ratios of primary endpoints. The difference between natural log-transformed mean serum *C*_max_ and AUC_inf_ for each treatment and the corresponding two-sided 90% CIs was calculated by ANOVA with allocation sequence, participants, administration period, and treatment as fixed effects. ANOVA, analysis of variance; AUC_inf_, area under the curve from 0 hours to infinity; CI, confidence interval; *C*_max_, maximum serum drug concentration; IP, investigational product; LS, least squares; PK, pharmacokinetic; SD, standard deviation.

PK equivalence was demonstrated between RGB-19 and tocilizumab for *C*_max_ and AUC_inf_, with GMRs for both outcomes within the predefined equivalence threshold of 0.80 to 1.25 (*C*_max_: point estimate: 1.04 [90% CI: 0.98-1.10]; AUC_inf_: point estimate: 1.05 [90% CI: 0.97-1.13]; Fig 2). Serum drug concentrations prior to the second IP administration were below the lower limit of quantification in all subjects, suggesting no evidence of carry-over between treatment periods.

There were no notable treatment differences in the mean or median values of secondary PK parameters (Table 2 and Supplementary Table S1). The secondary PK GMR point estimates all met the predefined equivalence threshold of 0.80 to 1.25, further demonstrating the similarity between treatments (Supplementary Table S2).

**Table 2:** PK parameters of serum drug concentration for the overall study period in the PK analysis set (*N* = 102)

	Treatment			
	RGB-19 (*N* = 102)		Tocilizumab (*N* = 102)	
AUC_last_ (μg × h/mL)				
Mean (SD)	2582.8 (873.8)		2502.9 (852.2)	
Median	2611.9		2487.1	
Range	465-4617		87-4262	
Geo. mean (GCV%)	2412.1 (41.6)		2287.6 (55.2)	
AUC_0-144_ (μg × h/mL)				
Mean (SD)	1167.8 (426.6)		1101.0 (369.1)	
Median	1180.1		1102.6	
Range	222-2097		88-1991	
Geo. mean (GCV%)	1080.7 (43.9)		1019.5 (47.4)	
AUC_144-t_ (μg × h/mL)				
N	102		101	
Mean (SD)	1415.0 (541.0)		1415.9 (556.9)	
Median	1371.9		1365.9	
Range	244-2846		28-2742	
Geo. mean (GCV%)	1299.6 (46.7)		1263.4 (63.9)	
*t*_max_ (h)				
Mean (SD)	108.305 (29.579)		111.470 (26.760)	
Median	107.5		107.7	
Range	47.57-172.30		48.00-172.63	
Geo. mean (GCV%)	–		–	
*t*_1/2_ (h)				
Mean (SD)	33.70 (20.06)		32.98 (17.01)	
Median	29.54		29.41	
Range	22.0-192.7		7.6-182.7	
Geo. mean (GCV%)	31.52 (30.6)		31.08 (31.7)	


PK values in ADA-positive/negative participants				
	RGB-19 (*N* = 102)		Tocilizumab (*N* = 102)	
	Positive	Negative	Positive	Negative
*C*_max_ (μg/mL)				
n	80	22	80	22
Mean (SD)	10.94 (3.41)	11.72 (3.76)	10.76 (3.07)	10.18 (3.48)
Median	11.20	11.25	11.00	9.20
Range	2.6-18.1	6.4-19.7	1.4-17.8	2.8-16.3
Geo. mean (GCV%)	10.33 (37.4)	11.16 (33.1)	10.19 (38.3)	9.52 (41.6)
AUC_inf_ (μg × h/mL)				
*n*	80	22	80	22
Mean (SD)	2499.1 (850.1)	2904.3 (909.4)	2506.4 (829.3)	2507.8 (957.7)
Median	2501.1	2904.7	2647.9	2355.6
Range	468-4619	1213-4534	88-4266	594-3882
Geo. mean (GCV%)	2330.8 (42.3)	2752.6 (36.1)	2289.1 (57.1)	2300.4 (48.4)

ADA, antidrug antibody; AUC_0-144_, area under the curve from 0 hours (immediately before administration) to 144 hours after administration; AUC_144-t_, area under the curve from 144 hours after administration to the last quantifiable time; AUC_inf_, area under the curve from 0 hours (immediately before administration) to infinity; AUC_last_, area under the curve from 0 hours (immediately before administration) to the last quantifiable time; Geo., geometric; GCV, geometric coefficient of variation; *N*, number of participants; *n*, number of participants per category; PK, pharmacokinetic; SD, standard deviation; *t*_1/2_, elimination half-life; *t*_max_, time to maximum serum concentration.

### PDs

**Figure 3:**
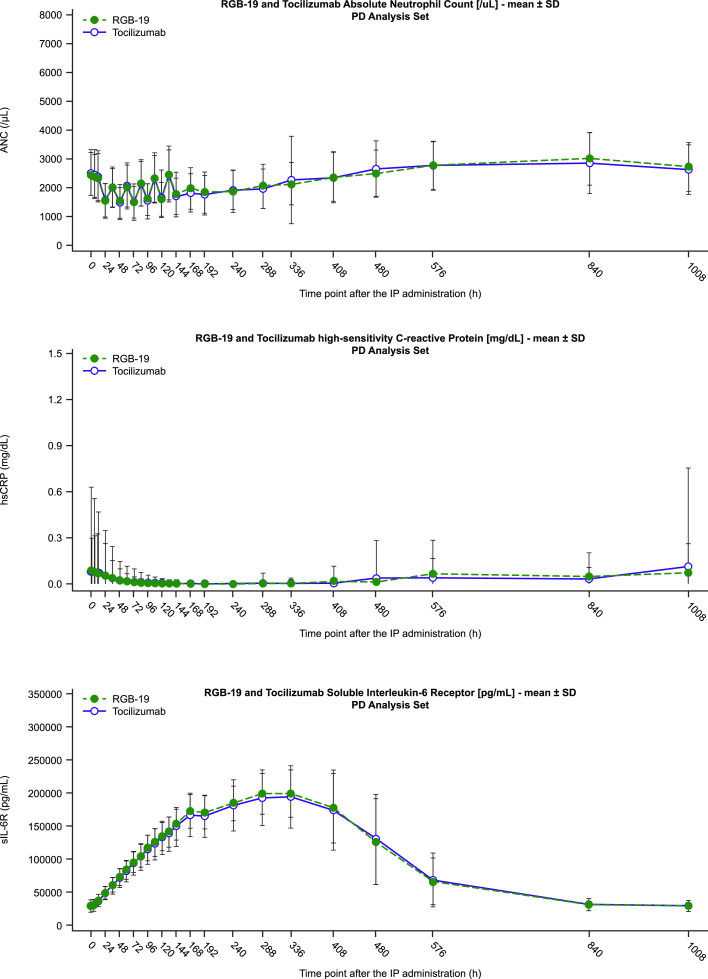
PD evaluation measures concentration-time profiles from the PD analysis set (*N* = 102). ANC, absolute neutrophil count; hsCRP, high-sensitivity C-reactive protein; IP, investigational product; PD, pharmacodynamic; SD, standard deviation; sIL-6R, soluble interleukin-6 receptor.

The concentration-time profiles for the PD parameters assessed (ANC, hsCRP, and sIL-6R) were generally comparable between groups for the overall time period. All PD parameters returned to baseline after 1008 hours (Fig 3).

The mean (SD) ANC AUEC values were −9688.0 (671,552.0) h/μL for RGB-19 and −96,000.3 (781,791.5) h/μL for tocilizumab. For RGB-19, the mean (SD) hsCRP AUEC values were −51.8 (529.7) mg × h/dL compared with −44.5 (205.4) mg × h/dL for tocilizumab. Mean (SD) sIL-6R AUEC values were 71,410.9 (18,750.4) ng × h/mL for RGB-19 and 70,303.2 (23,085.2) ng × h/mL for tocilizumab. The median values of the tested PD parameters were similar between groups (Supplementary Table S3).

### Safety

In total, 51 (47.2%) participants experienced 76 TEAEs after receiving RGB-19 and 53 (51.0%) participants experienced 78 TEAEs after receiving tocilizumab (Table 3). There were 36 (33.3%) participants receiving RGB-19 and 45 (43.3%) of participants receiving tocilizumab who experienced ADRs. No AEs resulting in death, serious ADRs/TEAEs, or severe ADRs/TEAEs were reported during the study.

**Table 3:** Summary of adverse events for the overall study period from participants administered IP (*N* = 110)

	Treatment					
	RGB-19 (*N* = 108)		Tocilizumab (*N* = 104)		Total (*N* = 110)	
	*n* (%)	e	*n* (%)	e	*n* (%)	e
**TEAEs**^a^	51 (47.2)	76	53 (51.0)	78	70 (63.6)	154
**AEs leading to study withdrawal or death**						
AEs resulting in study withdrawal	3 (2.8)	4	0 (0.0)	0	3 (2.7)	4
AEs resulting in death	0 (0.0)	0 (0.0)	0 (0.0)	0 (0.0)	0 (0.0)	0
**Severe AEs**	0 (0.0)	0 (0.0)	0 (0.0)	0 (0.0)	0 (0.0)	0
**ADRs, *n***	36 (33.3)	45	45 (43.3)	59	55 (50.0)	104
ADRs resulting in study withdrawal	2 (1.9)	2	0 (0.0)	0	2 (1.8)	2
ADRs resulting in death	0 (0.0)	0 (0.0)	0 (0.0)	0 (0.0)	0 (0.0)	0
**Severe ADRs**	0 (0.0)	0 (0.0)	0 (0.0)	0 (0.0)	0 (0.0)	0
**Serious ADRs**	0 (0.0)	0 (0.0)	0 (0.0)	0 (0.0)	0 (0.0)	0
**AEs reported in ≥5% of participants overall**						
Neutrophil count decreased	27 (25.0)	28	36 (34.6)	36	43 (39.1)	64
Upper respiratory tract infection	5 (4.6)	5	5 (4.8)	5	9 (8.2)	10

ADR, adverse drug reaction; AE, adverse event; e, number of events; IP, investigational product; *N*, number of participants; *n*, number of participants reporting at least one TEAE; TEAE, treatment-emergent adverse event.

a TEAEs starting after IP administration in period 1 were assigned to the treatment in period 1; TEAEs starting after IP administration in period 2 were assigned to the treatment in period 2.

Participants in both groups experienced decreased neutrophil count TEAEs (RGB-19: 28 events in 27/108 [25.0%] participants; tocilizumab: 36 events in 36/104 [34.6%] participants) (Table 3). However, there were no notable differences observed between groups in the mean ANC over time (Fig 3) or the incidence of infections and infestations between groups (RGB-19: 10 events in 9/108 [8.3%] participants; tocilizumab: 7 events in 7/104 [6.7%] participants). No participants experienced injection site tenderness or induration/swelling. Two (1.9%) participants experienced injection pain 1 hour after RGB-19 administration, 1 (0.9%) of whom also experienced injection pain at 2 hours after RGB-19 administration. Twelve hours after tocilizumab administration, 1 (1.0%) participant experienced injection site erythema/redness. All injection site reactions were of mild severity and were deemed to be ADRs by the investigator, and all resolved. There were no reports of diverticulitis or perforations during the study.

### Immunogenicity

Predose ADA positivity was observed in 3.6% (4/110) of participants overall. The number of ADA-positive and NAb-positive participants in the RGB-19 group was 61 (58.1%) and 44 (41.9%) from 105 active participants, respectively, compared with 62 (60.8%) and 48 (47.1%) from 102 active participants, respectively, in the tocilizumab group for the overall study period. ADA and NAb positivity by time point are presented in Supplementary Table S4. The mean difference in *C*_max_ and AUC_inf_ was assessed in ADA-positive/negative participants, with no clinically significant effects of immunogenicity on PK (Table 2).

## DISCUSSION

This phase 1 crossover study demonstrated PK equivalence and similarity in PD, safety profile, and immunogenicity between RGB-19 and tocilizumab. The GMR of primary PK parameters fell between the predefined equivalence margin of 0.80 to 1.25, confirming equivalence between RGB-19 and tocilizumab. This equivalence threshold is in line with the National Institute of Health Sciences, US Food and Drug Administration, and European Medicines Agency guidance for determining equivalence with biosimilars [[26], [27], [28]]. Similar results were seen across secondary PK parameters, further supporting equivalence between RGB-19 and tocilizumab.

No new safety signals were identified, and AE profiles were similar between treatments with no serious AEs or severe AEs observed. Decreased neutrophil counts were the most common AE. Changes in neutrophil counts are a well-known consequence of treatment with anti–IL-6 therapy [29,30], with similar reductions in neutrophil counts reported for tocilizumab and its biosimilars [18,31,32]. It has been shown that whilst neutropenia is associated with IL-6 inhibition, this does not affect neutrophil functions associated with host defence [33]. Indeed, there were no severe or serious infections reported in either group in this study.

Overall, the change in PD parameters over time was similar between treatments, with all PD parameters returning to baseline after 1008 hours. Although variability in the mean ANC was noted, the median ANC was similar between treatments. Overall, there were no clinically meaningful between-drug differences observed as a result of decreased neutrophil count in this study.

The incidence of ADAs and NAbs was similar between groups, with no clinical impact of immunogenicity on safety, regardless of treatment. Furthermore, there were no differences in the PK parameters *C*_max_ and AUC_inf_ between ADA-positive and ADA-negative participants. Taken together, these results show that immunogenicity had no effect on outcomes in this study.

Alternative doses and formulations of tocilizumab are available for use in other indications. IV tocilizumab is used for the treatment of indications such as cytokine release syndrome, polyarticular juvenile idiopathic arthritis, systemic juvenile idiopathic arthritis, and COVID-19, whilst SC tocilizumab is used for indications such as RA and giant cell arteritis [18,34]. This study included outcomes in participants who underwent SC administration of RGB-19 and tocilizumab. Data on outcomes in participants treated with an IV formulation of RGB-19 will be available in an upcoming phase 3 study (jRCT2031220512).

This was a phase 1 trial in healthy volunteers. As such, the study included a restricted participant population of males of Japanese race/ethnicity. However, these results are considered to be generalisable to other populations [18].

### Conclusions and implications

This study demonstrates the equivalence of RGB-19 and reference tocilizumab, showing that both treatments exhibit comparable PK, PD, immunogenicity, and safety outcomes. The study builds on previous research that has demonstrated the functional similarity of RGB-19 and tocilizumab. These phase 1 findings are expected to be further supported by efficacy findings from a phase 3 study in which participants with RA and an inadequate response to methotrexate were treated with IV RGB-19 or tocilizumab. Taken together, as a biosimilar with comparable outcomes to tocilizumab, RGB-19 is expected to offer a more cost-effective option that may help improve patient access to tocilizumab treatment.

## CRediT authorship contribution statement

**Tomoko Hasunuma:** Writing – review & editing, Supervision, Methodology, Investigation, Formal analysis, Conceptualization. **Paul Emery:** Writing – review & editing, Formal analysis. **Ernest Choy:** Writing – review & editing. **Masato Okada:** Writing – review & editing, Conceptualization. **Roshan Dias:** Writing – review & editing. **Károly Horvát-Karajz:** Writing – review & editing, Methodology, Formal analysis, Conceptualization. **Gordana Dancer:** Writing – review & editing. **Attila Kónya:** Writing – review & editing, Formal analysis. **Yusuke Karibe:** Writing – review & editing, Supervision, Project administration. **Kazuya Uchida:** Writing – review & editing. **Suguru Masuda:** Writing – review & editing, Formal analysis. **Joachim Kiefer:** Writing – review & editing, Methodology, Conceptualization. **Gerd R. Burmester:** Writing – review & editing.

## Competing interests

TH has nothing to declare. PE has provided expert advice to AbbVie, Activa, AstraZeneca, BMS, Boehringer Ingelheim, Galapagos, Gilead, Immunovant, Janssen, Lilly, and Novartis and contributed to clinical trials of AbbVie, BMS, Lilly, Novartis, and Samsung. EC has received research grants, speaking fees, consultancies, or honoraria from AbbVie, Bio-Cancer, Biocon, Biogen, Chugai Pharma, Eli Lilly, Fresenius Kai, Galapagos, Gedeon Richter, Gilead, Janssen, Pfizer, Sanofi, UCB, and Viatris. MO has received speaking fees and/or honoraria from Astellas, Eli Lilly and Company, GSK, and Janssen. RD, KH-K, GD, AK, and JK are Gedeon Richter employees. YK, KU, and SM are Mochida Pharmaceutical employees. GRB has received honoraria for lectures and consulting from Celltrion, Chugai, Fresenius, Gedeon Richter, and Sanofi. Given his role as editor-in-chief, GRB had no involvement in the peer review of this article and has no access to information regarding its peer review. Full responsibility for the editorial process for this article was delegated to another journal editor.

## Appendix Supplementary materials

Image, application 1

Image, application 2

## Footnotes

Supplementary material associated with this article can be found in the online version at doi:10.1016/j.ero.2025.08.006.
