# Supplementary material for: Comparison of the PK, PD, safety, tolerability, and immunogenicity of proposed biosimilar RGB-19 and tocilizumab in healthy Japanese males: a phase 1, randomised, crossover study
Source: EULAR Rheumatol Open. 2025 Sep 24;1(3):282–90. doi: 10.1016/j.ero.2025.08.006 (PMC13292239; doi:10.1016/j.ero.2025.08.006)
Supplement: Supplementary file 1 [file mmc1.pdf]

Participants randomised in Period 1		
Total	Sequence A	Sequence B
110	55	55

Participants who completed Period 1		
Total	Sequence A	Sequence B
102	49	53

Participants who withdrew from Period 1			
Main reason for withdrawal	Total	Sequence A	Sequence B
	8	6	2
Inclusion and exclusion criteria	0	0	0
Adverse events	3	3	0
Withdrawal by participant	0	0	0
Prohibited concomitant therapies	0	0	0
Positive drug or alcohol test at the day before IP administration in Period 2	1	0	1
Other reasons	4	3	1

Participants administered IP in Period 2		
Total	Sequence A	Sequence B
102	49	53

Participants who completed Period 2		
Total	Sequence A	Sequence B
102	49	53

Participants who withdrew from Period 2			
Main reason for withdrawal	Total	Sequence A	Sequence B
	0	0	0
Inclusion and exclusion criteria	0	0	0
Adverse events	0	0	0
Withdrawal by participant	0	0	0
Prohibited concomitant therapies	0	0	0
Positive drug or alcohol test at the day before IP administration in Period 2	0	0	0
Other reasons	0	0	0
