# Supplementary material for: Comparison of the PK, PD, safety, tolerability, and immunogenicity of proposed biosimilar RGB-19 and tocilizumab in healthy Japanese males: a phase 1, randomised, crossover study
Source: EULAR Rheumatol Open. 2025 Sep 24;1(3):282–90. doi: 10.1016/j.ero.2025.08.006 (PMC13292239; doi:10.1016/j.ero.2025.08.006)
Supplement: Supplementary file 2 [file mmc2.docx]

## Supplementary methods

*Full exclusion criteria*

- *Participants who had or had a history of serious infectious diseases, chronic or recurrent infections requiring treatment with anti-infective drugs or hospitalisation, diverticulitis, intestinal perforation, interstitial pneumonia and/or malignant tumour.*
- *Participants who had a history of tuberculosis (TB) infection, active TB, or latent TB infection based on clinical symptoms, chest X-ray tests, and interferon-γ release assays.*
- *Participants who were previously exposed to tocilizumab or any other IL-6 inhibitors or
  IL-6R inhibitors.*
- *Participants who, at any time of the study, had or had a history of cardiac, hepatic, renal, pulmonary, haematologic, gastrointestinal, thyroid, psychoneurological, metabolic/electrolyte, infectious disease or other disorders that were not suitable for this study in the opinion of the investigator(s).*
- *Participants with abnormal values in haematology tests, blood biochemistry tests, blood coagulation tests, or urinalysis during screening (deviation from the upper or lower limit of the reference range), excluding physiological change.*
- *Participants with a positive immunological test: Hepatitis B surface (HBs) antigen, HBs antibodies, hepatitis B core (HBc) antibodies, hepatitis C virus (HCV) antibodies, syphilis seroreactivity, or HIV antigens and antibodies.*
- *Participants with drug allergies, drug hypersensitivity or a history of hypersensitivity to the active substance or to any of the excipients of the IP.*
- *Patients who received a live vaccine 12 weeks prior to IP administration in Period 1 or were scheduled to receive a live vaccine during the study period.*
- *Participants who used prescription drugs 14 days prior to IP administration in Period 1 or over-the-counter drugs, pharmacist intervention required medicines or supplements (products containing one or more nutrients such as vitamins, minerals, herbs, amino acids and fatty acids in a form other than ordinary foods, such as tablets, capsules, powders and liquids) within 7 days prior to IP administration.*
- *Participants who suffered from or had a history of alcohol or drug abuse.*
- *Participants with a positive urine drug or alcohol test the day before administration in
  Period 1.*
- *Participants who consumed alcohol within 2 days of administration in Period 1.*
- *Smokers who smoked over 100 total cigarettes or smoked over a period of 6 months and had been smoking over the past month on the day of informed consent or those who had a positive smoking test result the day before administration in Period 1.*
- *Participants who underwent whole-blood collection of at least 200 mL within 4 weeks or at least 400 mL within 12 weeks prior to administration in Period 1, underwent blood component collection within 2 weeks prior to administration in Period 1, or underwent blood transfusion within 3 months prior to IP administration in Period 1.*
- *Participants who underwent whole-blood collection of at least 650 mL cumulatively outside of this study between 1 year prior to the day of informed consent and the day before IP administration in Period 1 and participants.*
- *Participants who received another IP within 16 weeks or five half-lives of the IP, whichever was longer, prior to the day of IP administration in Period 1.*
- *Participants who were not suitable for this study for any other reason in the opinion of the investigator(s).*

*List of Institutional Review Boards and Independent Ethics Committees*

| Country | Address |
| --- | --- |
| Japan | Review Board of Human Rights and  Ethics for Clinical Studies  Institutional Review Board  2-2-1, Kyobashi, Chuo-ku, Tokyo,  Japan |

## Supplementary results

Concomitant drugs for the treatment or prevention of adverse events were as follows, described using the Anatomical Therapeutic Chemical level 4 classification where possible: other drugs for functional gastrointestinal disorders (n=1 participant); opium alkaloids and derivatives, benzodiazepine derivatives and expectorants (n=1 participant); other antihistamines for systemic use (n=1 participant); propionic acid derivatives, other drugs for peptic ulcer and gastro-oesophageal reflux disease (GORD), opium alkaloids and derivatives, other antihistamines for systemic use, leukotriene receptor antagonists and other antivirals (n=1 participant); antihistamines (uncoded), corticosteroids for systemic use (combinations), potent corticosteroids (group III), other emollients and protectives, glucocorticoids, and agents for dermatitis excluding corticosteroids (n=1 participant); anti-inflammatory preparations, non-steroids for topical use, and other drugs for peptic ulcer and GORD (n=1 participant); penicillins with extended spectrum, propionic acid derivatives and amides (n=1 participant); neuraminidase inhibitors and anilides (n=1 participant); third-generation cephalosporins and macrolides (n=1 participant); anilides, other drugs for peptic ulcer and GORD, opium alkaloids and derivatives and neuraminidase inhibitors (n=1 participant); anilides, other throat preparations, mucolytics and opium alkaloids and derivatives (n=1 participant).

## Supplementary tables and figure

*Supplementary Table 1. Secondary PK parameters for the overall period from the PK analysis set (N=102)*

|  | **Treatment** | |
| --- | --- | --- |
|  | **RGB-19 (N=102)** | **Tocilizumab (N=102)** |
| V_d_/F (L) |  |  |
| Mean (SD) | 4.07 (6.24) | 4.37 (8.81) |
| Median | 2.75 | 2.78 |
| Min-Max | 1.3-51.0 | 1.6-87.9 |
| Geo. mean (GCV%) | 3.05 (63.0) | 3.17 (63.1) |
| CL/F (mL/h) |  |  |
| Mean (SD) | 73.70 (41.88) | 92.76 (180.59) |
| Median | 61.99 | 65.09 |
| Min-Max | 35.1-346.3 | 38.0-1846.6 |
| Geo. mean (GCV%) | 67.06 (41.5) | 70.70 (55.1) |
| k_el_ (/h) |  |  |
| Mean (SD) | 0.0227 (0.0048) | 0.0233 (0.0083) |
| Median | 0.0235 | 0.0236 |
| Min-Max | 0.004-0.031 | 0.004-0.091 |
| Geo. mean (GCV%) | 0.0220 (30.6) | 0.0223 (31.7) |
| MRT (h) |  |  |
| Mean (SD) | 171.65 (21.71) | 172.39 (26.41) |
| Median | 172.54 | 176.89 |
| Min-Max | 130.8-230.9 | 69.0-226.5 |
| Geo. mean (GCV%) | 170.29 (12.7) | 170.09 (17.4) |
| AUC_last_/AUC_inf_ (%) |  |  |
| Mean (SD) | 99.84 (0.20) | 99.83 (0.23) |
| Median | 99.89 | 99.89 |
| Min-Max | 98.4-100.0 | 98.0-100.0 |
| Geo. mean (GCV%) | 99.84 (0.2) | 99.83 (0.2) |

AUC_inf_, area under the curve from 0 hours (immediately before administration) to infinity; AUC_last_, area under the curve from 0 hours (immediately before administration) to the last quantifiable time; CL/F; apparent total clearance; Geo., geometric; GCV, geometric coefficient of variation; k_el_, elimination rate constant; MRT, mean residence time; N, number of participants; n, number of participants per category; PK, pharmacokinetic; SD, standard deviation; V_d_/F, volume of distribution.

*Supplementary Table 2. LS mean and GMR of secondary PK parameters for the overall period from the PK analysis set (N=102)*

| **Parameter** | **LS mean*** | | **GMR (N=99)^†^** | |
| --- | --- | --- | --- | --- |
|  | **RGB-19  (N=102)** | **Tocilizumab (N=102)** | **Point estimate** | **Two-sided  90% CI** |
| AUC_last_  (μg × h/mL) | 7.783 | 7.737 | 1.0469 | 0.9710 ～ 1.1288 |
| AUC_0-144_ (μg × h/mL) | 6.982 | 6.929 | 1.0534 | 0.9754 ～ 1.1377 |
| AUC_144-t_ (μg × h/mL) | 7.164 | 7.139 | 1.0253 | 0.9479 ～ 1.1091 |
| t_max_ (h) | 108.307 | 111.574 | N/A | N/A |
| t_1/2_ (h) | 3.450 | 3.435 | 1.0155 | 0.9387 ～ 1.0986 |
| V_d_/F (L) | 1.120 | 1.150 | 0.9701 | 0.8762 ～ 1.0740 |
| CL/F (mL/h) | 4.211 | 4.256 | 0.9553 | 0.8861 ～ 1.0299 |
| k_el_ (/h) | -3.817 | -3.801 | 0.9848 | 0.9103 ～ 1.0654 |
| MRT (h) | 5.136 | 5.136 | 1.0005 | 0.9779 ～ 1.0237 |

*Natural log-transformed values were used with the exception of t_max_, which used untransformed values. **^†^**GMR equals exp[difference in LS means (log-transformed scale)].
AUC_0-144_, area under the curve from 0 hours (immediately before administration) to 144 hours after administration; AUC_144-t_, area under the curve from 144 hours after administration to the last quantifiable time; AUC_last_, area under the curve from 0 hours (immediately before administration) to the last quantifiable time; CL/F, apparent total clearance; CI, confidence interval; CL/F, apparent total clearance; exp, exponential; GMR, geometric mean ratio; k_el_, elimination rate constant; LS, least square; MRT, mean residence time; N, number of participants, PK, pharmacokinetic; t_1/2_, elimination half-life; t_max_, time to maximum serum concentration; V_d_/F, volume of distribution.

*Supplementary Table 3. PD parameters for the overall period in the PD analysis set (N=102)**

|  | **Treatment** | |
| --- | --- | --- |
|  | **RGB-19 (N=102)** | **Tocilizumab (N=102)** |
| ANC AUEC (h/μL) |  |  |
| Mean (SD) | -9688.0 (671552.0) | -96000.3 (781791.5) |
| Median | 47738.5 | -43774.6 |
| Range | -3580430 to 1181313 | -3293483 to 1995680 |
| hsCRP AUEC (mg x h/dL) |  |  |
| Mean (SD) | -51.8 (529.7) | -44.5 (205.4) |
| Median | -5.8 | -8.0 |
| Range | -5307 to 361 | -1211 to 446 |
| sIL-6R AUEC (ng x h/mL) |  |  |
| Mean (SD) | 71410.9 (18750.4) | 70303.2 (23085.2) |
| Median | 69190.8 | 68852.5 |
| Range | 22897 to 126130 | -3814 to 144452 |

*Negative range values indicate a decrease relative to baseline.

AUEC, area under the effect-time curve; ANC, absolute neutrophil count; AUEC, area under the effect-time curve; hsCRP, high-sensitivity C-reactive protein; N, number of participants; n, number of participants per category; PD, pharmacodynamic; SD, standard deviation; sIL-6R, soluble interleukin-6 receptor.

*Supplementary Table 4. Immunogenicity by time point and period in the immunogenicity analysis set (N=110)*

| **Parameter** | **Finding** | **Period 1** | | | | | |
| --- | --- | --- | --- | --- | --- | --- | --- |
|  |  | **Day 1 pre-IP** | | **Day 13** | | **Day 43** | |
|  |  | **RGB-19 n/N (%) (Seq A)** | **Tocilizumab n/N (%) (Seq B)** | **RGB-19 n/N (%) (Seq A)** | **Tocilizumab n/N (%) (Seq B)** | **RGB-19 n/N (%) (Seq A)** | **Tocilizumab n/N (%) (Seq B)** |
| ADA | Positive | 2/55 (3.6) | 2/55 (3.6) | 2/53 (3.8) | 7/55 (12.7) | 26/52 (50.0) | 23/53 (43.4) |
|  | Negative | 53/55 (96.4) | 53/55 (96.4) | 51/53 (96.2) | 48/55 (87.3) | 26/52 (50.0) | 30/53 (56.6) |
| NAb | Positive | 0/2 (0.0) | 0/2 (0.0) | 1/2 (50.0) | 5/7 (71.4) | 18/26 (69.2) | 16/23 (69.6) |
|  | Negative | 2/2 (100.0) | 2/2 (100.0) | 1/2 (50.0) | 2/7 (28.6) | 8/26 (30.8) | 7/23 (30.4) |
| **Parameter** | **Finding** | **Period 2** | | | | | |
|  |  | **Day 43 pre-IP** | | **Day 55** | | **Day 85** | |
|  |  | **RGB-19 n/N (%) (Seq B)** | **Tocilizumab n/N (%) (Seq A)** | **RGB-19 n/N (%) (Seq B)** | **Tocilizumab n/N (%) (Seq A)** | **RGB-19 n/N (%) (Seq B)** | **Tocilizumab n/N (%) (Seq A)** |
| ADA | Positive | 23/53 (43.4) | 26/52 (50.0) | 23/53 (43.4) | 28/49 (57.1) | 35/53 (66.0) | 39/49 (79.6) |
|  | Negative | 30/53 (56.6) | 26/52 (50.0) | 30/53 (56.6) | 21/49 (42.9) | 18/53 (34.0) | 10/49 (20.4) |
| NAb | Positive | 16/23 (69.6) | 18/26 (69.2) | 21/23 (91.3) | 26/28 (92.9) | 26/35 (74.3) | 32/39 (82.1) |
|  | Negative | 7/23 (30.4) | 8/26 (30.8) | 2/23 (8.7) | 2/28 (7.1) | 9/35 (25.7) | 7/39 (17.9) |

Sequence A: RGB-19 on Day 1 followed by tocilizumab on Day 43; Sequence B: tocilizumab on Day 1 followed by RGB-19 on Day 43.
ADA, antidrug antibody; IP, investigational product; N, number of subjects; n, number of cases; NAb, neutralising antibody; Seq, sequence.

*Supplementary Figure 1. Participant disposition (N=110)*

IP, investigational product; N, number of participants.
